# Impact of CT scan parameters on deformable image registration accuracy using deformable thorax phantom

**DOI:** 10.1002/acm2.13917

**Published:** 2023-02-25

**Authors:** Ryutaro Ikeda, Noriyuki Kadoya, Yujiro Nakajima, Shin Ishii, Takayuki Shibu, Keiichi Jingu

**Affiliations:** ^1^ Department of Radiation Oncology Tohoku University Graduate School of Medicine Sendai Japan; ^2^ Department of Radiology Japanese Red Cross Ishinomaki Hospital Ishinomaki Japan; ^3^ Department of Radiological Science Komazawa University Tokyo Japan

**Keywords:** deformable image registration, lung, physical phantom, radiotherapy, target registration error

## Abstract

The purpose of this study was to evaluate the deformable image registration (DIR) accuracy using various CT scan parameters with deformable thorax phantom.

Our developed deformable thorax phantom (Dephan, Chiyoda Technol Corp, Tokyo, Japan) was used. The phantom consists of a base phantom, an inner phantom, and a motor‐derived piston. The base phantom is an acrylic cylinder phantom with a diameter of 180 mm, which simulates the chest wall. The inner phantom consists of deformable, 20 mm thick disk‐shaped sponges with 48 Lucite beads and 48 nylon cross‐wires which simulate the vascular and bronchial bifurcations of the lung. Peak‐exhale and peak‐inhale images of the deformable phantom were acquired using a CT scanner (Aquilion LB, TOSHIBA). To evaluate the impact of CT scan parameters on DIR accuracy, we used the four tube voltages (80, 100, 120, and 135 kV) and six reconstruction algorithms (FC11, FC13, FC15, FC41, FC44, and FC52). Intensity‐based DIR was performed between the two images using MIM Maestro (MIM software, Cleveland, USA). Fiducial markers (beads and cross‐wires) based target registration error (TRE) was used for quantitative evaluation of DIR.

In case with different tube voltages, the range of average TRE were 4.44–5.69 mm (reconstruction algorithm: FC13). In case with different reconstruction algorithms, the range of average TRE were 4.26–4.59 mm (tube voltage: 120 kV). The TRE were differed by up to 3.0 mm (3.96–6.96 mm) depending on the combination of tube voltage and reconstruction algorithm.

Our result indicated that CT scan parameters had moderate impact of TRE, especially for reconstruction algorithms for the deformable thorax phantom.

## 1 INTRODUCTION

In recent years, deformable image registration (DIR) method has been used in the field of radiotherapy. Applications of DIR enable dose accumulation,^1^, ^2^ automatic contour propagation,^3^ and computed tomography (CT) ventilation to indirectly evaluate lung function,^4^, ^5^ taking into account changes in physique and position changes during the treatment period by using this technology. However, there is uncertainty with regard to DIR, and it has been reported that the accuracy of DIR differs depending on the software used, and the institution using the technology.^6^ Therefore, the accuracy needs to be examined by each institution when using DIR. The American Association of Medical Physics (AAPM) published the TG132 guideline^7^ summarizing the use of image registration techniques including DIR, and image registration quality assurance (QA) methods; thus, we can say that the examination of the accuracy of DIR is an important matter. As one method of QA in DIR, it is recommended to perform QA using a physical moving phantom that is actually capable of deformation. Regarding the DIR QA with this phantom, we can obtain the actual deformation vector field using a landmark implemented in the phantom. Various studies have been conducted with regard to phantoms used in this technique.^8^, ^9^

Many deformation algorithms applying image intensity are used in the deformation parameters of DIR.^10^ In the field of radiomics in which analyses are similarly performed using image intensity information,^10^, ^11^, ^12^ it has been reported that the quantity of features obtained changes according to the scan parameters used to obtain the CT image used,^13^ and Kim et al. reported that when different reconstruction algorithms are used a predominant difference occurs in 19 features calculated.^14^

Therefore, similarly in DIR also, it is conceivable that the accuracy will be affected by changing the parameters that affect image intensity information when performing CT scans. However, the impact of CT scan parameters on DIR accuracy has not been elucidated. Yet, tests cannot be performed using images of actual human bodies, and consequently tests must be performed using phantoms. Therefore, in the present study, we used a dynamic deformable phantom to test the extent by which DIR accuracy is affected by tube voltage and reconstruction algorithm, which are CT scans parameters that affect image quality.

## 2 METHODS

### 2.1 Deformable thoracic phantom

**FIGURE 1:**
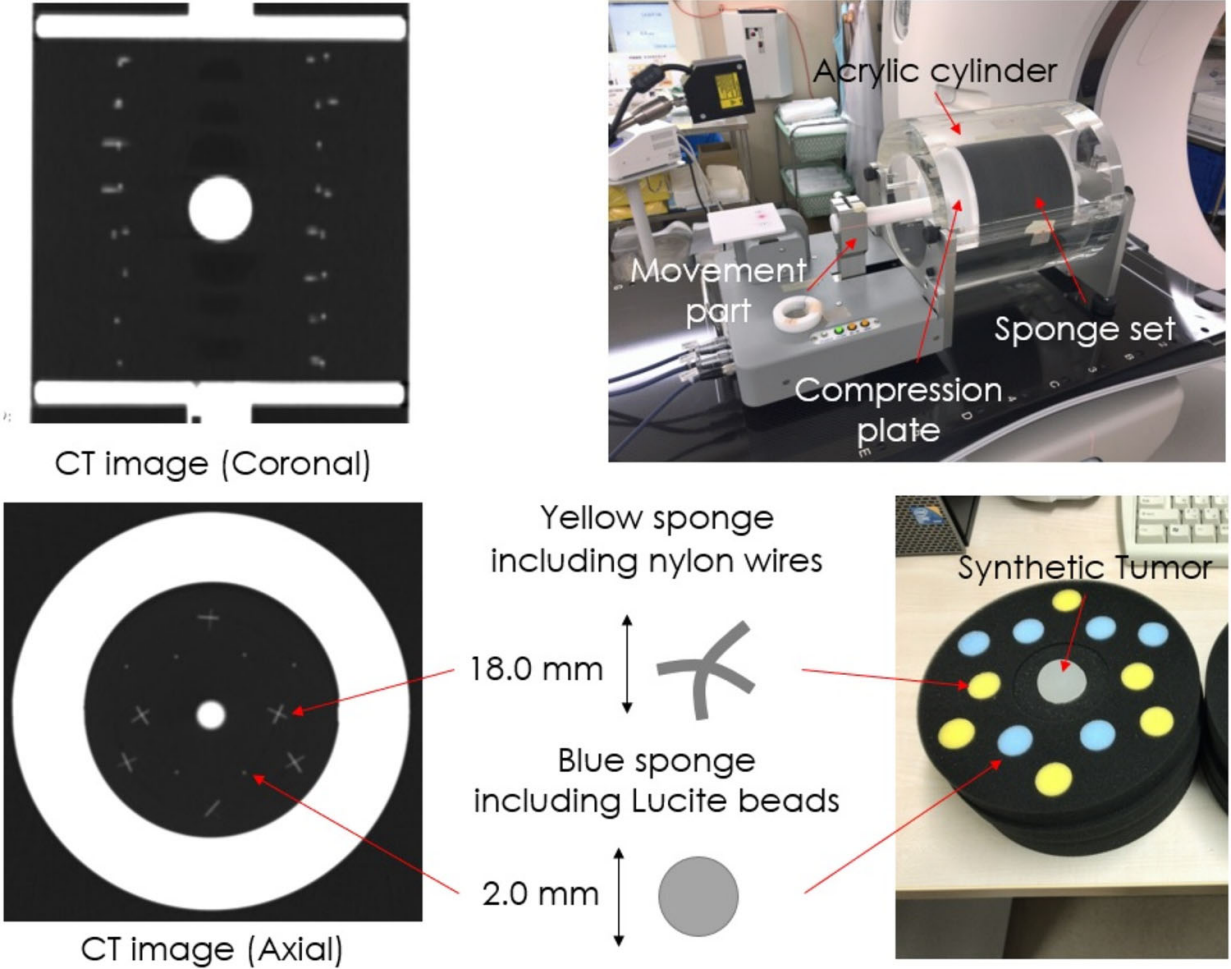
Overview and deformable thorax phantom.

The dynamic deformable phantom (Dephan, Chiyoda Technol Corp, Tokyo, Japan) consists of a base phantom, an inner phantom, and a motor‐derived piston. An overview of our phantom is shown in Figure 1. The base phantom was an acrylic cylinder phantom 180 mm in diameter, which simulates the chest wall. The inner phantom consists of deformable disk‐shaped sponges. Disk‐ shaped sponges are made of a soft material (physical density, 0.22 g/cm^3^) 175 mm in diameter and 20 mm thick. There are multiple holes (20.0 mmφ) in the disk‐shaped sponge, and embedding‐type cylinder‐shaped sponges, in which the Lucite beads (2.0 mmφ) or nylon cross‐wires (1.4 mmφ and 18.0 mm) were inserted, were incorporated into the hollowed‐out spaces in the disk‐shaped sponge. Furthermore, the soft tissue sphere (30.0 mmφ) can be inserted. This enables us to place many beads and nylon cross‐wires at multiple arbitrary points to simulate various inner phantom settings. The Lucite beads and nylon cross‐wires (48 nylon wires and 48 Lucite beads simulate the vascular and bronchial bifurcations of the lung. The piston, simulating the diaphragm, is fastened to a programmable motor via a metal rod that compresses and decompresses the lung according to an arbitrary, programmable breathing profile. The details are described in the article by Sugawara et al.^15^

### 2.2 Acquisition of peak‐exhale and peak‐inhale images using various scan parameter and reconstruction settings

Peak‐exhale and peak‐inhale CT scan images of the phantom were taken using a CT scanner (Aquilion LB, Canon Medical Systems, Tochigi, Japan) with different four scanning parameters (i.e., 80, 100, 120, and 135 kV). In general, 120 kV is preferred to use for treatment planning CT scan. To investigate the impact of tube voltage on DIR accuracy, we additionally used the uncommon scanning parameter (e.g., 80 kV). The tube current was set to 150 mA, and with an exposure time of 0.5 s, the mAs value was 75mAs. The slice thickness was set to 2 mm, and the voxel size was 0.98 mm × 0.98 mm × 2 mm. The volume CT dose index were 3.3 mGy (80 kV), 5.9 mGy (100 kV), 9.0 mGy (120 kV), and 11.6 mGy (135 kV), respectively. The peak‐exhale image was acquired at compression phase (the phantom settings of 20 mm) and the peak‐exhale image was acquired at decompression phase (the phantom settings of 0 mm).

In addition, to evaluate the impact of CT reconstruction algorithm, we used different six reconstruction algorithms (FC11, FC13, FC15, FC41, FC44, and FC 52). FC11 is a reconstruction function for abdominal/mediastinum conditions, and FC13 and 15 are functions of the same series, and the larger the number, the sharper the reconstruction function. FC41 and 44 are reconstruction functions for the head condition, and FC44 enables sharper reconstruction than FC41. FC52 is a reconstruction function for lung field conditions and is a function used for sharp image reconstruction. The details of the filters are not disclosed due to vendor business secrets.

### 2.3 Performing of deformable image registration

DIR was performed between peak‐exhale image and peak‐exhale image. We used the free‐form deformation‐based algorithm implemented in MIM ver.6.6 (Cleveland, OH, USA). The default smoothing factor was set to 0.5. We set the volume of interest (VOI) within the inner phantom not including the motor‐derived piston surrounding the phantom.

### 2.4 Evaluation of DIR accuracy

To assess the accuracy of DIR, we used the target registration error (TRE). Dice similarity coefficient is a widely acceptable method for evaluation of DIR accuracy. However, this method may not suitable in the case which the evaluation structure is extremely small (e.g., cross‐wire). Thus, we used the TRE in this study. We set 96 landmarks (48 points on wire intersections, and 48 points on markers) on both peak‐inhale and peak‐exhale images. To calculate TRE using landmarks, we applied the formula below.

(1) $$\mathrm{TRE}\;=\sqrt{{\left(x1-x2\right)}^{2}+{\left(y1-y2\right)}^{2}+{\left(z1-z2\right)}^{2}},$$

where, *x*1, *y*1, and *z*1 indicate the landmark coordinates on the fixed image, while *x*2, *y*2, and *z*2 indicate the landmark coordinates on the deformed image. The TRE was calculated for all landmarks, and the mean value of which was calculated.

To correlation between DIR accuracy and CT image quality, we calculated the standard deviation (SD) of CT value within cubic ROI which covered one cross‐wire at peak‐inhale image.^16^

In addition, we did the ANOVA statistical analysis to test the significant difference in TRE among all tube voltages or reconstruction algorithms using JMP software (version 16.2.0, SAS Institute Inc., USA) (significant: *p* < .05).

## 3 RESULT

**FIGURE 2:**
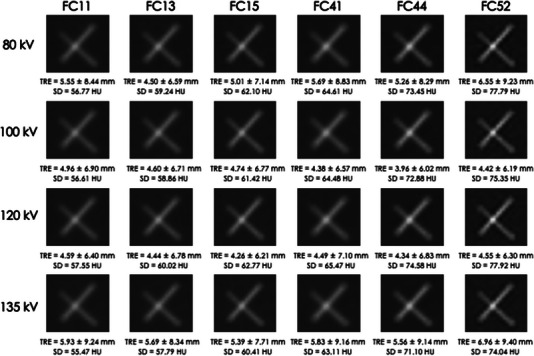
CT image obtained by different reconstruction algorithms and tube voltages.

**FIGURE 3:**
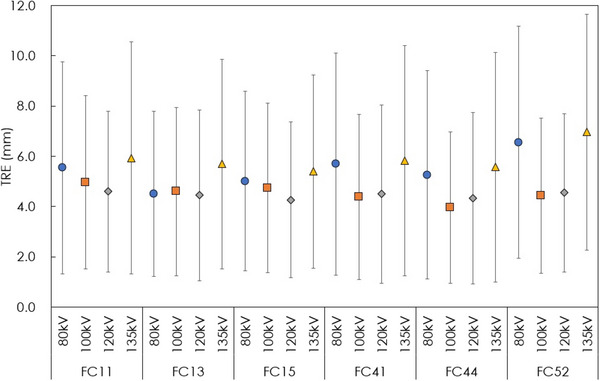
Summary of the DIR accuracy for each reconstruction algorithm and tube voltage.

Figure 2 shows images obtained with each reconstruction algorithm and tube voltage. We found that blurring and sharpness of markers visually change according to changes in tube voltage and reconstruction algorithm. Figure 3 presents a summary of the TRE according to all reconstruction algorithms and tube voltages combined. In case with different tube voltages, the range of average TRE were 4.59–5.93 mm (FC11), 4.44–5.69 mm (FC13), 4.26–5.39 mm (FC15), 4.38–5.83 mm (FC41), 3.96–5.56 mm (FC44), and 4.42–6.96 mm (FC52). In case with different reconstruction algorithms, the range of average TRE were 4.50–6.55 mm (80 kV), 3.96–4.96 mm(100 kV), 4.26–4.59 mm (120 kV), and 5.39–6.96 mm (FC52). The TRE differed by up to 3.0 mm (3.96–6.96 mm) depending on the combination of tube voltage and reconstruction algorithm. The significance differences were observed among tube voltages (*p* = 0.002) but they were not observed among reconstruction algorithms (*p* = 0.609).

**FIGURE 4:**
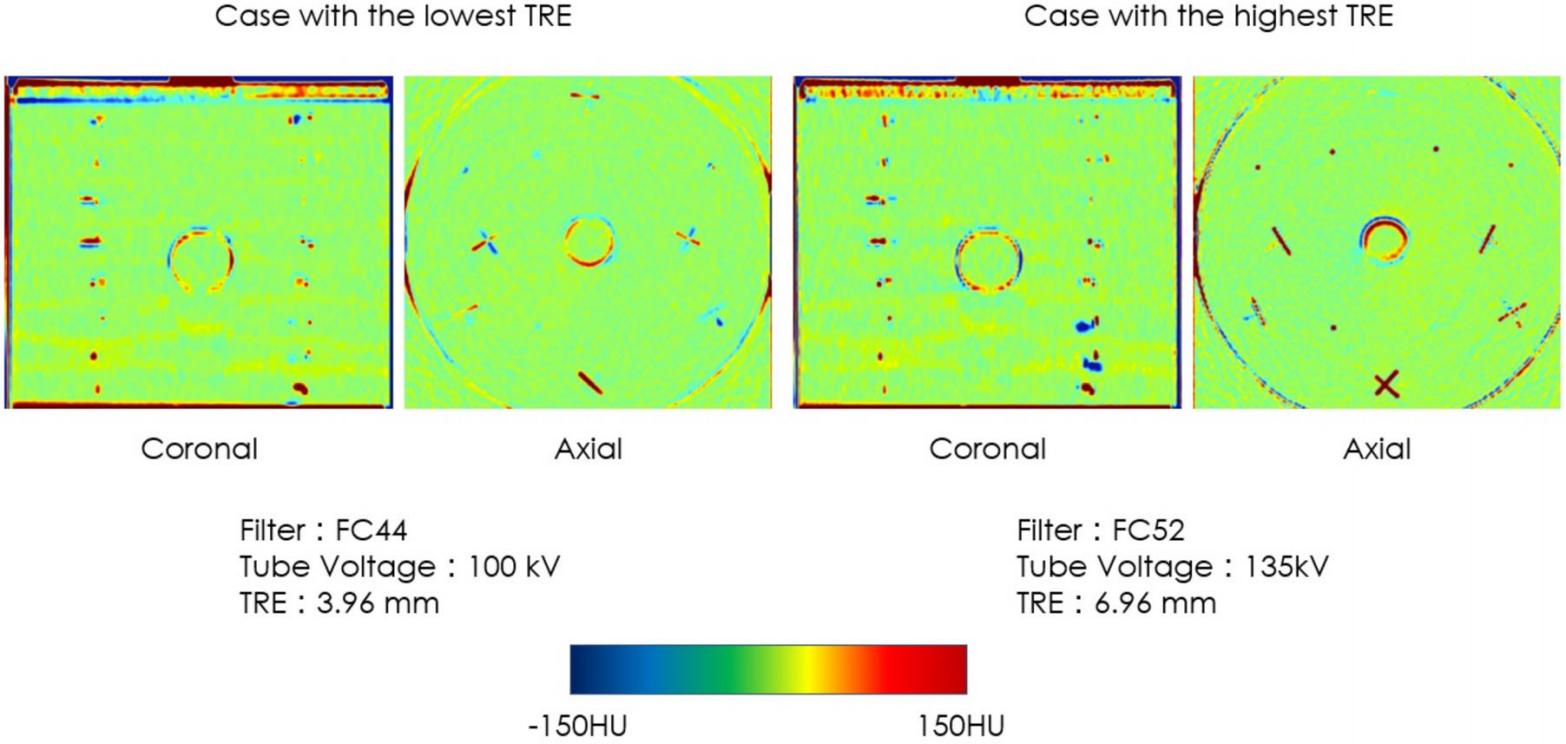
Difference images between the fixed image and deformed image for the typical two cases (case with the highest TRE and the case with the lowest TRE).

**FIGURE 5:**
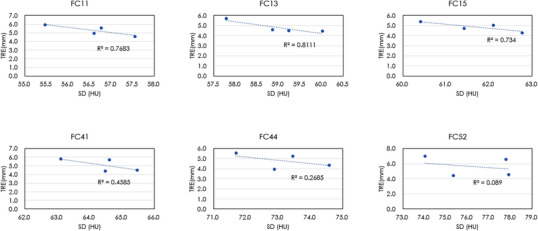
The relationship between SD and TRE for each reconstruction algorithm.

**FIGURE 6:**
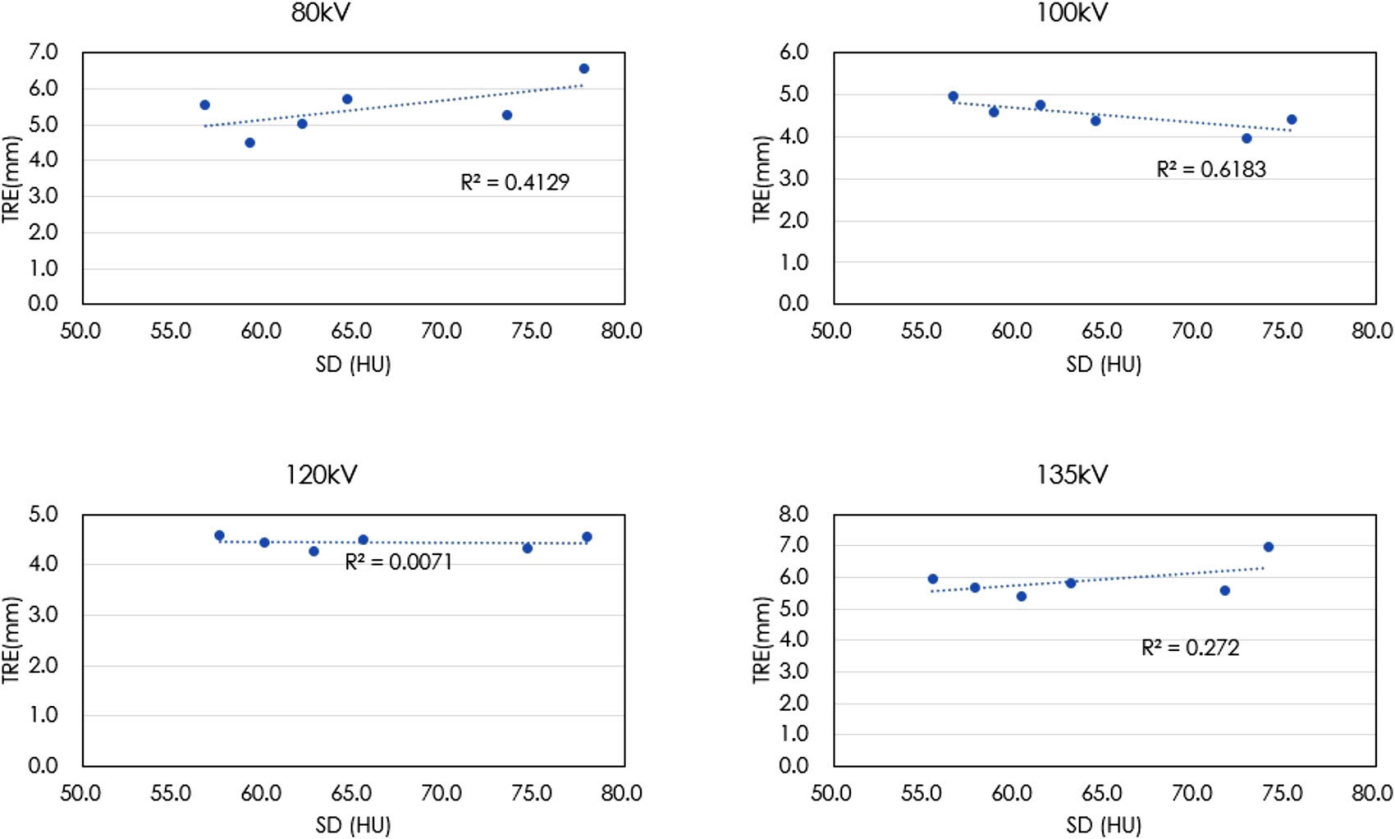
The relationship between SD and TRE for each tube voltage.

Figure 4 shows the difference images of the fixed image and deformed image in typical two cases (the case with the highest TRE and the case with the lowest TRE). In the case with the highest TRE, deformed image showed more deformation compared to the fixed image. Figures 5 and 6 show the correlation between SD and TRE when the tube voltage or reconstruction algorithm was constant. Depending on the tube voltage, a negative strong correlation between the SD and TRE, or a positive strong correlation was observed.

## 4 DISCUSSION

In the present study, we examined how the accuracy of DIR is affected by CT scan parameters. Our result showed that the TRE changed from 4.56 to 4.59 mm due to reconstruction algorithm. In addition, the TRE changed from 4.44 to 5.69 mm by changing the tube voltage. This result suggested that DIR accuracy between peak‐exhale and peak‐inhale images may be changed due to combination of tumor voltage and reconstruction algorithm.

Regarding the reconstruction algorithm, our result showed that the TRE changed from 4.56 to 4.59 mm due to the algorithms. Different reconstruction algorithms caused the different amount of blurring for CT image. The DIR accuracy is reduced in blurred images. Davis et al. showed the highest HU differences (e.g., 56 HU for density to water and 117 HU for bone‐like materials) in CT images when changing reconstruction algorithm using Toshiba Aquilion LB scanner which was used in our study.^17^ Their result is consistent with our result. In addition, we found the FC52 algorithm caused the largest difference in the TRE. Since this algorithm is created for lung regions, this has a higher image resolution. On the other hand, the image noise was emphasized. This can be explained by the fact that the images with FC52 had higher SD values around markers. This emphasized noise may hinder corresponding anatomical points between fixed and moving images during DIR process, resulting in lower DIR accuracy.

Regarding the tube voltages, the tube voltages changed the TRE in all reconstruction algorithms (e.g., FC13, TRE:4.44–5.69 mm). Overall, the TRE tended to decrease between 80 and 120 kV and the TRE increased for 135 kV. The reason for this may be that increased x‐ray intensity, which caused by the increased tube voltage, reduced the image contrast. Konishi et al. reported that increasing the tube voltage increased the x‐ray intensity,^18^ and that increasing the number of x‐ray photons that reach the detector created images with less noise. However, they reported that high tube voltage for CT scans reduces the difference in the linear attenuation coefficient of the material. Due to this effect, our result with 135 kV may have the lower DIR accuracy (e.g., higher TRE). That is, the difference in the linear attenuation coefficient between the marker and sponge used in the phantom became smaller, resulting in lower contract.

Our result suggested that the DIR accuracy may be affected by CT scan parameters. The AAPM TG 132 task report describes the DIR evaluation (e.g., commissioning and regularly QA for DIR) method using a physical phantom, virtual phantom, and actual patient images.^7^, ^19^ When the virtual phantom and actual patient images were used, we can check the impact of the DIR software configuration and DIR parameter setting on DIR accuracy, but we cannot check the impact of CT scanning parameters on DIR accuracy. On the other hand, physical phantom can do the end‐to‐end test for DIR accuracy including the impact of CT scanning parameters.

To achieve higher DIR accuracy, we may optimize the suitable reconstruction algorithm and tube voltage. However, in clinical practice, since we basically determine the optimized reconstruction algorithms and tube voltage based on the clinical impact, it may be difficult to optimize the reconstruction algorithm and tube voltage for DIR performance. Samavati et al. reported that the hybrid algorithm improved the DIR performance (e.g., the TRE by up to 1 mm).^12^ When you cannot optimize the reconstruction algorithm and tube voltage for DIR accuracy, the optimization for DIR method can be useful for improvement of the DIR accuracy.

In addition, Samavati et al. reported that 1.6 mm of TRE caused the 1 Gy dose error of DIR‐based total accumulated dose for minimum dose.^20^ Our result showed that the combination of the reconstruction algorithm and tube voltage caused the large difference (TRE >3 mm). Based on the result reported by Samavati et al., we estimated that this TRE difference caused uncertainty of 1 Gy or more in the total accumulated dose.

The present study has a limitation. First, we used only one CT scanner. We are scheduled to use the different CT scanner. Next, we used the only one DIR software program (e.g., MIM Mestro). Different DIR algorithm implanted in different DIR software (e.g., Velocity, and RayStation) may have different result. Second, the phantom which we used is designed for thoracic region and reproduces the only high contrast tissue. Other tissue type with low contrast (e.g., parotid) may have the different impact of CT scan parameters on DIR accuracy. Further evaluation is needed to clearly this. Third, we only evaluated the two main CT scan parameters (i.e., tube voltage and reconstruction algorithm). In general, the two CT scan parameters (i.e., tube voltage and reconstruction filter) are relatively easy to change in acquisition of treatment planning CT images. Thus, we used these two CT scan parameters.

There are other CT scan parameters (e.g., pitch and CT slice thickness). These parameters may have impact for DIR accuracy. In the near future, we will investigate the different other imaging parameters. Finally, The TRE values were relatively large compared to those that can be found in the literature.^6^, ^7^ This may due to usage of simple physical phantom which cannot provide the rich information about surrounding tissues for DIR. In addition, the reason for this may be that in both fixed and moving images, FOV for DIR was mostly occupied by air, so there was little information to optimally perform the DIR.

## 5 CONCLUSION

We evaluated the effect of CT image scan parameters on DIR accuracy (TRE). Our result indicated that CT scan parameters had moderate impact of TRE, especially for reconstruction algorithms for the deformable thorax phantom which had the high contrast tissue. The TRE were differed by up to 3.0 mm (3.96–6.96 mm) depending on the combination of tube voltage and reconstruction algorithm.

## AUTHOR CONTRIBUTIONS

RI and NK contributed to the conception and design of the study. RI and YN wrote the program. RI and NK performed the analysis. RI mainly drafted the manuscript. NK, YN, SI, TS, and KJ reviewed the manuscript. All authors read and approved the final manuscript.

## CONFLICT OF INTEREST STATEMENT

The authors declare no conflict of interest with regard to this manuscript.
